# GCWOAS2: Multiobjective Task Scheduling Strategy Based on Gaussian Cloud-Whale Optimization in Cloud Computing

**DOI:** 10.1155/2021/5546758

**Published:** 2021-06-10

**Authors:** Lina Ni, Xiaoting Sun, Xincheng Li, Jinquan Zhang

**Affiliations:** ^1^College of Computer Science and Engineering, Shandong University of Science and Technology, Qingdao 266590, China; ^2^Key Laboratory of the Ministry of Education for Embedded System and Service Computing, Tongji University, Shanghai 201804, China

## Abstract

An important challenge facing cloud computing is how to correctly and effectively handle and serve millions of users' requests. Efficient task scheduling in cloud computing can intuitively affect the resource configuration and operating cost of the entire system. However, task and resource scheduling in a cloud computing environment is an NP-hard problem. In this paper, we propose a three-layer scheduling model based on whale-Gaussian cloud. In the second layer of the model, a whale optimization strategy based on the Gaussian cloud model (GCWOAS2) is used for multiobjective task scheduling in a cloud computing which is to minimize the completion time of the task via effectively utilizing the virtual machine resources and to keep the load balancing of each virtual machine, reducing the operating cost of the system. In the GCWOAS2 strategy, an opposition-based learning mechanism is first used to initialize the scheduling strategy to generate the optimal scheduling scheme. Then, an adaptive mobility factor is proposed to dynamically expand the search range. The whale optimization algorithm based on the Gaussian cloud model is proposed to enhance the randomness of search. Finally, a multiobjective task scheduling algorithm based on Gaussian whale-cloud optimization (GCWOA) is presented, so that the entire scheduling strategy can not only expand the search range but also jump out of the local maximum and obtain the global optimal scheduling strategy. Experimental results show that compared with other existing metaheuristic algorithms, our strategy can not only shorten the task completion time but also balance the load of virtual machine resources, and at the same time, it also has a better performance in resource utilization.

## 1. Introduction

With the rapid development of Internet of Things and big data technology, cloud computing [1] also occupies the most important position in business interconnection [2]. Users do not need to master the underlying application technology to use various resources in the cloud platform on demand [3, 4]. Cloud computing provides users with a more convenient way to use, and more and more people have noticed the cloud platform. Cloud computing integrates and manages a large number of idle resources through the Internet and manages and redistributes them. At the same time, users use the cloud platform to send tasks to the data center of the cloud terminal. Therefore, cloud computing systems are faced with hundreds of millions or even billions of jobs that need to be scheduled every day. The number of tasks is very large, and each job occupies a certain amount of cloud system resources [5]. Cloud resources are heterogeneous, dynamic, and limited. Therefore, under the premise of satisfying user QoS, it is the field of cloud computing to efficiently schedule massive tasks and allocate cloud resources reasonably so that cloud tasks occupy as few cloud resources as possible [6]. The challenging issue is also a common concern of scholars today.

The scheduling problem of cloud computing is mainly divided into three layers: the first is scheduling virtual resources for user applications [7], the second is scheduling virtual resources (virtual machines) to physical resources, and the third is the scheduling and landing of physical resources. These kinds of scheduling have multiple different goals to be optimized at each layer, so the scheduling problem in cloud computing is extremely complex, and it is considered as an NP-hard problem. In this article, we focus on studying the first layer of cloud computing scheduling, which is creating a VM to allocate resources reasonably for tasks sent by users [8]. At this layer, tasks are placed in virtual machines for execution. In the process of task execution, scheduling technology is particularly important. It affects the operating efficiency of the entire system, user service quality, system load balancing, system energy consumption, etc. [9–11]. Therefore, at this level, we must design an appropriate scheduling strategy to solve the above problems.

In the cloud computing environment, many forms of scheduling methods have been adopted, including priority-based scheduling [12–14], cluster-based scheduling [15, 16], QoS-based scheduling [17, 18], multiprocessor-based scheduling [19], and heuristic algorithm-based scheduling [20]. We can use exhaustive scheduling algorithms to meet scheduling requirements when the task request is small and the scale of cloud computing is also small. However, as the scale of the problem becomes larger, the dimensions and the variables we need to optimize are increasing, and exhaustive algorithms will be destroyed by dimensions. At this time, heuristic algorithms began to receive more and more attention. The heuristic algorithm is more efficient than the blind search algorithm. Although the optimal solution cannot be obtained for the NP-hard problem, the heuristic algorithm can approach the optimal solution infinitely and obtain a relatively optimal solution. In recent years, various heuristic algorithms have been used in scheduling problems in various fields, including production scheduling and cloud computing task scheduling. Summarizing the previous research, most of them still use the EC algorithm based on the overall [21], for example, the cloud computing task scheduling which is based on genetic algorithm [22], ant colony algorithm [23], and particle swarm optimization algorithm [24, 25]. These algorithms can effectively use the information exchange in the group to achieve the purpose of global search.

Among EC algorithms, the whale optimization algorithm (WOA) [26] is a new heuristic algorithm that simulates the predation behavior of humpback whales. Compared with other heuristic optimization algorithms, the whale optimization algorithm (WOA) uses random and search agents to simulate the hunting behavior of humpback whales and the spirals to simulate the attack behavior of the bubble net, and it updates the position information of the humpback whales in these two ways in order find the best position of the humpback whale. This algorithm has a simple search strategy, strong search ability, and fast convergence speed. When applied to the cloud computing task scheduling process, it can quickly converge to obtain the most suitable scheduling plan. However, the convergence accuracy of WOA is very low, and the algorithm can easily fall into a local optimum. In the entire task scheduling process, it is very likely that the local optimal solution will be found, and the resulting scheduling plan is not the best plan among all choices.

The cloud model [27] is an uncertain transformation model that deals with qualitative concepts and quantitative descriptions, which includes forward cloud generators and reverse cloud generators. It has universality, randomness, and fuzziness and can realize the conversion of fuzzy concepts to specific data. The Gaussian cloud model can reflect the randomness of the concept by using the Gaussian distribution and the universality of the Gaussian membership function. However, the normal Gaussian cloud model is not very effective in finding the optimal solution of the problem, so applying it to the task scheduling process cannot find the most suitable scheduling scheme. Inspired by bionics and cloud generators, in this article, we consider combining the whale optimization algorithm (WOA) with the Gaussian cloud model and a multiobjective task scheduling strategy based on Gaussian cloud-whale optimization (GCWOAS2) is proposed for task scheduling in cloud computing.

In this article, our main purpose is to find a reasonable mapping between tasks and virtual machines to reduce execution time and reduce costs while meeting user's QoS. Therefore, we designed a three-layer scheduling model to achieve multiobjective scheduling and optimization. In the GCWOAS2 strategy, taking into account the blindness of the randomly initialized population, the opposition-based learning (OBL) mechanism [26] is introduced. In this way, the fitness of this individual and the opposing individual can be obtained, and individuals with less fitness can be selected from the two to form a new population, thereby obtaining a better population than randomly generated individuals, and further obtaining a better scheduling plan in the initial stage. In addition, we also introduce an adaptive learning factor to make the search step in this strategy adaptive. In this way, the search process can dynamically change according to the environment, which also enables the user's task to search for the VM in a larger range during the scheduling process. In addition, the Gaussian cloud model is introduced in the whale's swimming process, and a multiobjective task scheduling algorithm based on the Gaussian whale-cloud optimization-GCWOA algorithm is proposed. This algorithm not only has the randomness and universality of Gaussian cloud model, but also the random search function can also be applied to the WOA algorithm under the guidance of qualitative knowledge. In the process of multiobjective optimization, the scheduling process is more random, the task will be easier to find a suitable virtual machine and generate a suitable scheduling plan, and it can also make the algorithm jump out of the local optimum as soon as possible to avoid premature convergence.

In this paper, we study the multiobjective task scheduling problem based on the Gaussian whale-cloud model in cloud computing. The main contributions are summarized as follows:

1. A three-layer architecture model of whale-Gauss cloud scheduling is proposed, which are user task layer, task scheduling layer, and data center layer to describe the entire process of task scheduling.
2. In the population initialization process, an initialization population strategy based on the opposition-based learning mechanism is proposed to generate the scheduling plan, so that the initialized scheduling plan is closer to the optimal plan. Simulation experiments show that each individual in the new population is closer to the optimal solution.
3. An adaptive adjustment factor based on dynamic changes is proposed to dynamically adjust the search step size to make the task search for VMs in a wider range in the scheduling process. Experiments have proved that this strategy can get more solutions than before.
4. A whale optimization algorithm based on the Gaussian cloud model is proposed, which makes the scheduling process more random and fuzzier, can make the scheduling strategy jump out of the local optimum, and enhances the optimization ability, with significant effects.
5. Finally, a multiobjective task scheduling algorithm based on the Gaussian whale-cloud optimization-GCWOA algorithm is proposed, and the GCWOA algorithm is used in the GCWOAS2 scheduling strategy, which can effectively combine all the advantages of the above contributions.

The other parts of this article are arranged as follows: in the second part, we study the related work. In the third part, we describe the task scheduling model. In the fourth part, the basic knowledge is introduced. In the fifth part, we propose our own method. The sixth part compares and evaluates our models. And the seventh part draws a summary.

## 2. Related Work

The heterogeneity and dynamics of cloud resources make the scheduling strategy for effectively allocating resources to tasks particularly complicated. In addition, the CPU running rate and bandwidth of virtual machines and hosts in the cloud are different, which will cause scheduling problems becoming more difficult.

### 2.1. Heuristic Algorithm Task Scheduling

To make more reasonable and effective use of resources in the cloud, metaheuristic algorithms [28] have begun to be gradually applied to cloud computing and are developing rapidly. Scholars at home and abroad began to propose many task scheduling schemes based on metaheuristic algorithms, for example, scheduling algorithms based on GA, PSO, and ACO.

Xiong et al. [29] analyzed the scheduling problem on the cloud data center, combined Johnson's algorithm with the genetic algorithm, designed a two-stage task scheduling, and optimized the makespan of each virtual machine. In order to reasonably arrange the problem of virtual machine placement under data-intensive services, in [30], Wang et al. proposed an improved particle swarm optimization algorithm, which weighs the energy consumption of the task and the global QOS guarantee. Zhao et al. [31] combined Bayes' theorem and clustering process to find the best cluster of hosts, which improves system throughput and achieves overall load balancing.

Ding et al. [32] proposed a two-stage energy-saving cloud computing method based on Q-learning to reduce task response time and improve resource utilization. In [33], Sanaj and Joe Prathap proposed a MAP reduction framework and GA-WOA algorithm to schedule tasks in the cloud, and obtained a more efficient task scheduler. In [34], Dhinesh Babu and Venkata Krishna proposed a heuristic algorithm based on bee behavior to maximize throughput and achieve load balancing of virtual machines. Chen et al. [35] proposed an improved WOA algorithm to improve the convergence speed and accuracy in the scheduling process and improve resource utilization.

However, the scheduling focus of these methods is on the optimal solution of a single goal, and the overall situation cannot be considered. Although this example achieves the best results, it will lead to the effects of other examples are not very well, so that the results have great limitations.

### 2.2. Multiobjective Task Scheduling

With the application of cloud computing in the industrial Internet, single-objective optimization can no longer meet industrial needs, and multiobjective optimization has begun to attract more and more attention. Many scholars have conducted a lot of research in the context of multiobjective scheduling. The current research on multiobjective optimization mainly includes task completion time, QoS quality, operating cost, and energy consumption [36].

Hosseinzadeh et al. [37] conducted extensive research on multiobjective optimization tasks and workflow scheduling, classified and compared the multiobjective optimization algorithm schemes, and obtained the advantages and limitations of each scheme. Mohammadzadeh et al. [38] proposed a hybrid multiobjective greedy ALO algorithm based on chaos theory. The algorithm is optimized for multiobjective workflow scheduling problems to reduce manufacturing time and operating costs and improve throughput.

In [39], Xu et al. proposed a new multiobjective task scheduling model to obtain a suitable task allocation strategy. Peng et al. [40] proposed an online resource scheduling framework based on the deep Q-network (DQN) algorithm. The purpose is to resolve the conflict between cloud service providers who want to minimize energy costs and customers seeking to optimized services reducing energy consumption and task manufacturing. Huang et al. [41] considered multiobjective planning in cloud computing task scheduling and proposed a multiobjective particle swarm optimization to increase the search capability of the feasible solution space in the algorithm and enhance the global and local search capabilities.

In evolutionary computation, balancing the diversity and convergence of the population for multiobjective evolutionary algorithms (MOEAs) is one of the most challenging topics [42]. In the project scheduling problem, in order to solve the problem of limited multiskill tasks, Wang and Zheng [43] proposed a knowledge-guided multiobjective fruit fly optimization algorithm (MOFOA) to minimize the manufacturing cycle and total cost. In Ali et al.'s study [44], to minimize manufacturing time and total cost in a foggy cloud environment, a multiobjective task scheduling optimization model based on the discrete nondominated sorting genetic algorithm II (DNSGA-II) is proposed. Kumari et al. [45] designed a super heuristic resource scheduling based on the multiobjective particle swarm and genetic algorithm to improve resource utilization and throughput and reduce manufacturing time.

Although the above method has good performance in multiobjective scheduling, it has insufficient advantages in the convergence speed and convergence accuracy of the algorithm, and further optimization is needed.

In order to solve the above problems, in this paper we propose a multiobjective task scheduling strategy based on Gaussian cloud-whale optimization (GCWOAS2). A good scheduling strategy can effectively reduce the response time of tasks, meet the needs of users with different constraint levels, and further improve resource utilization and reduce energy consumption. So far, we have not found any research on applying the WOA algorithm to cloud task scheduling. Therefore, we apply the GCWOAS2 strategy proposed to cloud task scheduling in this paper. Experiments have proved that this can effectively reduce task execution time and balance the load.

## 3. System Model

In cloud computing, scheduling strategies can directly affect the utilization efficiency of resources at all levels and the operating costs of service providers, so the quality of task scheduling becomes the key to the smooth and convenient completion of production operations [46].

### 3.1. Whale-Gaussian Cloud Scheduling Model

**Figure 1:**
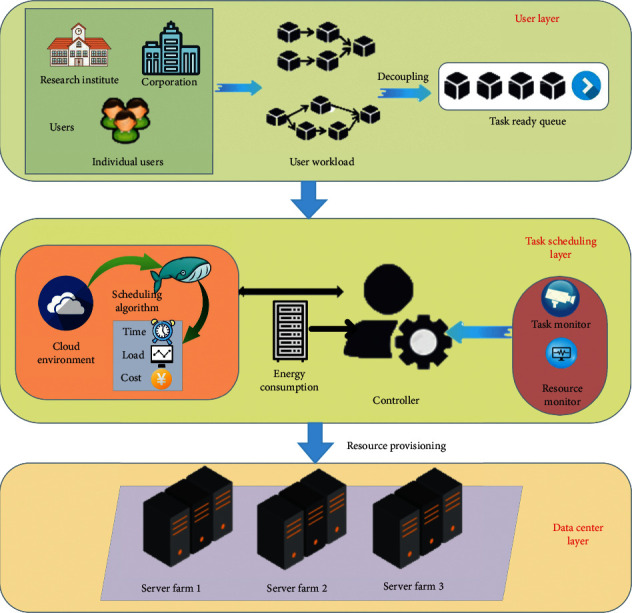
Whale-Gaussian cloud scheduling model architecture.

In this article, we designed a whale-Gaussian cloud scheduling model, as shown in Figure 1. The model is composed of three layers, which are user task layer, task scheduling layer, and data center layer. The details in the system model are as follows:

1. User task layer: in this layer, after the user sends a task, a series of workloads are split into subtasks and placed in the task waiting queue so that the tasks can be scheduled in the next layer.
2. Task scheduling layer: in this layer, task monitors and resource managers feed back the remaining tasks and resource information to the control center, and the control center feeds back the information to the nodes in the cloud environment. Tasks are scheduled on the computing nodes according to our proposed GCMWOA algorithm. After the scheduling is completed, the task and resource information is fed back to the control center. In this way, nodes in the cloud can cooperate and communicate to complete the user's task request.
3. Data center layer: in this layer, the virtual machine information is sent to the server, and the scheduling and landing of physical resources are completed in the server.

The demand information submitted by the user is transformed into a set of subtasks at the task level. These subtasks enter the task waiting queue. At the scheduling layer, tasks begin to find suitable resource nodes in the cloud environment. The model can clearly divide all processes of task scheduling and make the scheduling of each part more intuitive. However, the scheduling strategy and scheduling algorithm we proposed are mainly concentrated on the second layer of the model-task scheduling layer. In this process, we assume that all tasks are independent of each other.

When we use algorithms to schedule tasks to virtual machines for execution, since it is unknown which virtual machine the task is suitable for running in, many solutions will be randomly generated when the algorithm initializes the population, so we propose to introduce the opposition-based learning mechanism to the initialization In the population, when the population is initialized, the generated solution will be closer to the optimal solution. We have introduced an adaptive dynamic adjustment factor, so that our proposed algorithm will be able to search for as many tasks and virtual machine matching schemes as possible, which is conducive to obtaining the optimal solution. Since the algorithm may save the local suboptimal solution as the optimal solution in the iterative process, in order to avoid this problem, we introduce the Gaussian cloud model so that the algorithm can jump out of the local optimum and find the optimal solution.

### 3.2. Model Definition

To conveniently express the task scheduling process we designed, we give the following definitions:

**Definition 1 .**

Tasks and VM set: suppose there is a set of virtual machine resources recorded as {*M*_1_, *M*_2_,…, *M*_*m*_} and *N* > *M*. Any computing resource VM can be described as{id, cpu, mem, bw}, respectively, representing the virtual machine number, the execution speed, memory, and bandwidth of the virtual machine, and the unit is generally MIPS. The task is described as {id, length}, which, respectively, represent the task number and task length.

**Definition 2 .**

Mapping matrix A between tasks and VMs: each task in Definition 1 can only run on one virtual machine. One virtual machine can handle multiple tasks. All tasks are independent of each other. The correspondence between virtual machines is represented by a matrix as follows:

(1) $$\begin{array}{c} A_{nm}=\left[\begin{array}{c} a_{11},a_{12},\ldots ,a_{1m}\\ a_{21},a_{22},\ldots ,a_{2m}\\ \cdots \\ a_{n1},a_{n2},\ldots ,a_{nm} \end{array}\right]. \end{array}$$

We use *a*_*ij*_ to represent the mapping relationship between tasks and virtual machines. When *a*_*ij*_=1, it means that the third task is executed on the fourth virtual machine. For each *i*, we stipulate ∑_*j*=1_^*m*^*a*_*ij*_=1.

**Definition 3 .**

Time function: in this article, the length of the task set and the running speed of the virtual machine are known. Our goal is to optimize task completion time, balance virtual machine load, and reduce operating costs. The time can be calculated according to formula (2), as follows:

(2) $$\begin{array}{c} \text{ET}\left(i,j\right)=\frac{{\text{tlength}}_{i}}{{\text{vcpu}}_{j}},\;1\leq i\leq n,1\leq j\leq m, \end{array}$$

where ET(*i*, *j*) represents the time it takes for task *i* to execute on virtual machine *j*, tlength_*i*_ represents the length of task *i*, and vcpu_*j*_ represents the running speed of virtual machine *j*. The task completion time is the maximum time for the virtual machine to complete the task, expressed by formulas:

(3) $$\begin{array}{c} {\text{time}}_{j}=\sum_{j=1}^{m}a_{ij}\text{ET}\left(i,j\right), \end{array}$$

(4) $$\begin{array}{c} f_{1}=\max\left\{{\text{time}}_{1},{\text{time}}_{2},\ldots ,{\text{time}}_{m}\right\}, \end{array}$$

where time_*j*_ represents the sum of the execution time of all tasks on the virtual machine *j*(1 ≤ *j* ≤ *m*) and *f*_1_ represents the maximum value of the task execution time on the virtual machine.

**Definition 4 .**

Load cost function: load balancing means that the completion time of the virtual machine is basically the same as the total completion time of the task. We define the load balancing function as shown in the following formula:

(5) $$\begin{array}{c} f_{2}=a_{ij}\sqrt{\frac{\sum_{j=1}^{m}{\left({\text{vt}}_{j}-{\text{vavg}}_{j}\right)}^{2}}{m}}. \end{array}$$

In formula (5), vt_*j*_ represents the running time of the task on virtual machine *j*, that is, the load on virtual machine *j*. vavg_*j*_ represents the average execution time of tasks executed on virtual machine *j*, that is, the average load on virtual machine *j*. *f*_2_ is the smaller load of the virtual machine which is more balanced.

**Definition 5 .**

Operational cost function: the cost spent on a virtual machine is calculated by using the CPU, memory, and bandwidth of the virtual machine in this article. We define the cost function as shown in

(6) $$\begin{array}{c} f_{3}=a_{ij}\sum_{j=1}^{m}\left(v_{\text{cpuj}}\times P_{1}+v_{\text{bwj}}\times P_{2}+v_{\text{ramj}}\times P_{3}\right). \end{array}$$

In formula (6), *v*_cpuj_ represents the CPU operating rate of the *j*-th virtual machine, *v*_bwj_ represents the virtual machine bandwidth, and *v*_ramj_ represents the memory size of the *j*-th virtual machine.

In order to more accurately converge and get the optimal solution, we use the min-max method to normalize the matrix. This method is used for normalization because the numerical proportions of the three targets *f*_1_, *f*_2_, and *f*_3_ are quite different. In this case, the search for the optimal solution will be biased and the target with the largest proportion will be biased. It will affect the optimization accuracy.

**Definition 6 .**

Multiobjective function: express the above three objective functions as *F*_1_, *F*_2_, and *F*_3_, and normalize as shown in the following formulas:

(7) $$\begin{array}{c} F_{1}=\frac{f_{1}}{\max\left(f_{1}\right)}, \end{array}$$

(8) $$\begin{array}{c} F_{2}=\frac{f_{2}}{\max\left(f_{2}\right)}, \end{array}$$

(9) $$\begin{array}{c} F_{3}=\frac{f_{3}}{\max\left(f_{3}\right)}. \end{array}$$

Different cloud computing systems have different performance requirements for target tasks. Therefore, in this article, we convert the above three objective function utilization weights from multiobjective scheduling to single-objective scheduling. The final objective function becomes

(10) $$\begin{array}{c} F_{\text{obj}}=\min\left\{w_{1}F_{1}+w_{2}F_{2}+w_{3}F_{3}\right\}. \end{array}$$

In formula (8), the weight *w* can be adjusted according to the proportion according to the actual application requirements of the system. For example, if we pay more attention to optimizing time in the whole process, we can take *w*_1_=0.5, *w*_2_=0.25, and *w*_3_=0.25. At this time, a large number of tasks will be allocated to the VM with relatively high CPU running speed and relatively large memory to save time.

From the overall optimization of the algorithm, in order to obtain the smallest *F*_obj_, the larger the value of *w*_*i*_, the smaller the value of corresponding *F*_*i*_, which will result in different scheduling. Whether the task is allocated to a VM with a larger CPU or to a VM with a larger memory and resource bandwidth will be affected by the value of *w*_*i*_. This configuration of using weights to influence the optimization target will speed up the convergence speed of the algorithm. Here, we do not consider the extreme cases of *w*_1_=1, *w*_2_=0, and *w*_3_=0. This setting transforms the problem into single-objective scheduling.

**Definition 7 .**

Optimization objective function: in this article, the three objectives to be optimized are equally important, so we set *w*_1_=*w*_2_=*w*_3_. Based on this situation, we get the formula

(11) $$\begin{array}{c} F_{\text{obj}}=\min\left\{\frac{1}{3}F_{1}+\frac{1}{3}F_{2}+\frac{1}{3}F_{3}\right\}. \end{array}$$

In this article, our main optimization goals are task execution time, virtual machine load, and the cost of running all virtual machines. In the process of task scheduling, the commonly used method to reduce task execution time is to place the task in the virtual machine with the fastest running speed, that is, the largest CPU value, but this will cause the load of the virtual machine with a large CPU value to be too high. Other virtual machines with small CPU values are idle, resulting in unbalanced load, which in turn affects operating costs. This kind of operation is more efficient in terms of time, but it sacrifices the load of the virtual machine and also affects the cost. Therefore, we propose a multiobjective scheduling in this article. Our strategy can take into account the time and load issues at the same time, so that all objectives are optimized.

## 4. Preliminaries

In this part, we give the main ideas and algorithms of the whale optimization algorithm and Gaussian cloud model and use them as the basis for cloud computing task scheduling based on the GCMWOA algorithm.

### 4.1. Whale Optimization Algorithm (WOA)

Whale optimization algorithm (WOA) [26] is an algorithm proposed based on the behavior of whales in hunting prey. In this algorithm, the position of each whale represents a mapping between tasks and virtual machines in scheduling. In *D*-dimensional space, the position of the whale is *X*={*x*_1_, *x*_2_,…, *x*_*D*_}. In the process of hunting, whales will have two behaviors: one is to surround the prey and the other is to drive the prey with a bubble net. In the process of swimming, the whale will randomly choose these two behaviors to hunt down prey.

The probability of choosing whether to surround the prey or the bubble net to trap the prey is the same when the whale swims, that is, *P* (enclose) = *P* (bubble net) = 0.5. When a whale chooses to surround its prey, it will choose to move to the optimal position or to a random whale position.

#### 4.1.1. Surround Prey

When choosing to move to the optimal position,

(12) $$\begin{array}{c} \overset{⟶}{D}=\overset{⟶}{C}\times \overset{⟶}{X_{b}}\left(t\right)-\overset{⟶}{X}\left(t\right), \end{array}$$

(13) $$\begin{array}{c} \overset{⟶}{X}\left(t+1\right)=\overset{⟶}{X_{b}}\left(t\right)-\overset{⟶}{A}\times \overset{⟶}{D}, \end{array}$$

where $\overset{⟶}{D}$ represents the enclosing step size, *t* represents the current algebra, $\overset{⟶}{A}$ and $\overset{⟶}{C}$ are coefficient vectors, and $\overset{⟶}{X_{b}}$ represents the optimal position vector in the solution space, and the calculation formulas for coefficient vectors $\overset{⟶}{A}$ and $\overset{⟶}{C}$ are shown in

(14) $$\begin{array}{c} \overset{⟶}{A}=2\overset{⟶}{a}\times \overset{⟶}{r_{1}}-\overset{⟶}{a}, \end{array}$$

(15) $$\begin{array}{c} \overset{⟶}{C}=2\times \overset{⟶}{r_{2}}. \end{array}$$

In each iteration process, $\overset{⟶}{r_{1}}$and $\overset{⟶}{r_{2}}$ are random vectors between [0, 1]. As the convergence factor, $\overset{⟶}{a}$ decreases from 2 to 0 as the number of iterations. The change of $\overset{⟶}{a}$ is shown in the formula

(16) $$\begin{array}{c} \overset{⟶}{a}=2-\frac{2T}{T_{\max}}. \end{array}$$

When choosing to swim to the position of a random whale, the swimming formula is shown in the following formula:

(17) $$\begin{array}{c} \overset{⟶}{X}\left(t+1\right)=\overset{⟶}{X_{\text{rand}}}\left(t\right)-\overset{⟶}{A}\times \overset{⟶}{D}, \end{array}$$

where $\overset{⟶}{X_{\text{rand}}}$ is the random position of the whale in the solution space. The value of the coefficient vector $\overset{⟶}{A}$ determines how the whale swims during the enclosing phase. When $\overset{⟶}{A}<1$, move towards the optimal individual; when *n*$\overset{⟶}{A}>1$, move towards a random individual.

#### 4.1.2. Bubble Net Attack Prey

While whales use bubble nets to attack their prey, they also continuously update their position by directly constructing a spiral equation between themselves and their prey. The position update of the whale is shown in the following formulas:

(18) $$\begin{array}{c} \overset{⟶}{D^{\prime }}=\overset{⟶}{X_{b}}\left(t\right)-\overset{⟶}{X}\left(t\right), \end{array}$$

(19) $$\begin{array}{c} \overset{⟶}{X}\left(t+1\right)=\overset{⟶}{D^{\prime }}\times e^{bl}\times \;\cos\left(2\pi l\right)+\overset{⟶}{X_{b}}\left(t\right). \end{array}$$

In formula (19), *b* is a constant used to define the shape of a logarithmic spiral, and *l* is a random number uniformly distributed between [−1,1].

The task scheduling solution we proposed can be transformed into the whale position problem in the whale optimization algorithm [35]. The search process of whale swimming is the process of task finding a suitable virtual machine. The position of the leader whale is the optimal solution in the whole scheduling process, and the fitness function value of the leader is the minimum value of *F*_obj_. In this way, we can use the WOA algorithm to schedule cloud computing tasks to get an optimal solution. In each iteration, each whale must update its own position information according to the formula, put the position information into matrix *A*_*nm*_ for execution, calculate the fitness value of the whale at this position according to the fitness function *F*_obj_, and find the optimal position is used as the position of the leader whale for the next iteration. Repeat this process until the iteration ends, and finally find the optimal solution of the entire population in matrix *A*_*nm*_, that is, the best solution for performing scheduling.

### 4.2. Opposition-Based Learning

The concept of opposition-based learning (OBL) was proposed by Tizhoosh [47] in 2005. Its main idea is to generate reverse individuals based on existing individual information in the current space, so that reverse individuals participate in the competition with the current individual, compare the candidate solution and the reverse solution, and select a better individual to enter the iteration. Experiments prove that this mechanism is effective in improving the search efficiency of the algorithm.

Opposition-based learning is defined as follows: assuming that the solutions generated in *D*-dimensional space are *x*_*i*_=(*x*_*i*1_, *x*_*i*2_,…, *x*_*iD*_), *x*_*ij*_ ∈ [*lb*, *ub*], *lb* and *ub* are the upper and lower limits of the solution space, respectively. Suppose its reverse solution is *x*_*i*_′=(*x*_*i*1_′, *x*_*i*2_′,…*x*_*iD*_′), and the solution and reverse solution in the space satisfy the following formula:

(20) $$\begin{array}{c} x_{ij}^{\prime }=k\left({\text{lb}}_{ij}+{\text{ub}}_{ij}\right)-x_{ij}, \end{array}$$

where *k* can be real numbers with different values. When *k*=0, *x*_*i*_′ is a general OBL based on solving symmetry; when *k*=0.5, *x*_*i*_′ is a general OBL based on interval symmetry. Here, we use generalized OBL, taking *k*=1.

### 4.3. Gaussian Cloud Model

The cloud model [27] was proposed by Li Deyi et al. in 1995. It is an uncertainty conversion model between qualitative concepts and quantitative expressions expressed by linguistic values, which realizes the conversion between qualitative concepts and quantitative values. Cloud models have now been applied to various fields such as natural language processing and image processing. The cloud model has three numerical characteristics, which are expected *E*_*x*_, entropy *E*_*n*_, and superentropy *H*_*e*_, which are denoted as *C*=(*E*_*x*_, *E*_*n*_, *H*_*e*_). Expectation is the distribution expectation of cloud drops in space, which can represent a qualitative concept. Entropy is a measure of uncertainty in qualitative concepts, which can reflect randomness and ambiguity. The superentropy is the uncertainty measure of entropy, which is determined by the randomness and ambiguity of entropy.

The normal cloud model is an algorithm that basically obeys the normal distribution of cloud drops. After determining the expectations *E*_*x*_ and *H*_*e*_, normal random numbers and cloud drops can be calculated to calculate the degree of membership. The calculation formula is as follows:

(21) $$\begin{array}{c} E_{ni}^{\prime }=\text{Normrnd}\left(E_{n},H_{e}\right), \end{array}$$

(22) $$\begin{array}{c} x_{i}=\text{Normrnd}\left(E_{ni},\left|E_{ni}^{\prime }\right|\right), \end{array}$$

(23) $$\begin{array}{c} y_{i}=\exp\left[\frac{-{\left(x_{i}-E_{x}\right)}^{2}}{2{\left(E_{ni}^{\prime }\right)}^{2}}\right]. \end{array}$$

The pseudocode of the Gaussian cloud model generation algorithm is shown in (Algorithm 1).

**Figure 2:**
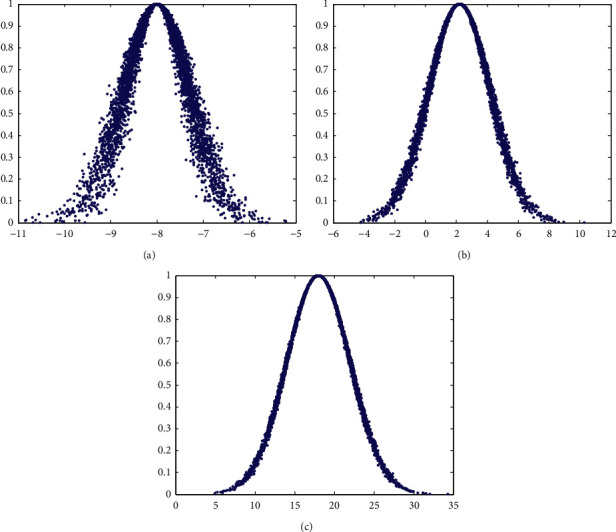
Gaussian cloud model under different conditions. (a) *E*_*x*_=−8,  *E*_*n*_=0.7, *H*_*e*_=0.1,  and *n*=5000. (b) *E*_*x*_=2.2,  *E*_*n*_=2, *H*_*e*_=0.1,  and *n*=5000. (c) *E*_*x*_=18,  *E*_*n*_=4*H*_*e*_=0.1,  and *n*=5000.

The distribution and digital characteristics of cloud drops in Gaussian clouds are shown in Figure 2.

Figure 2(a) represents a one-dimensional cloud model with expectation *E*_*x*_=−8, entropy *E*_*n*_=0.7, superentropy *H*_*e*_=0.1, and cloud drop number *n*=5000.  Figure 2(b) represents a cloud model with expectation *E*_*x*_=2.2, entropy *E*_*n*_=2, superentropy *H*_*e*_=0.1, and cloud drop number *n*=5000.  Figure 2(c) represents a one-dimensional cloud model with *E*_*x*_=18, entropy *E*_*n*_=4, superentropy *H*_*e*_=0.1, and cloud drop number *n*=5000. The digital characteristics of the cloud can be clearly seen through the three graphs in Figure 2.

## 5. Application of Whale Optimization Algorithm in Task Scheduling

The problem of searching for the best mapping between tasks and virtual machines in the process of task scheduling in cloud computing can be transformed into the problem of solving the optimal position of whales in the whale optimization algorithm. The search process of whale swimming is the process of task finding a suitable virtual machine. The position of the leader whale is the optimal solution in the entire scheduling process. The minimum value of the leader's fitness function value is the minimum value obtained by normalizing time, load, and cost in the entire scheduling process [34]. In this way, we can use the WOA algorithm to get an optimal solution in cloud computing task scheduling. In each iteration, each whale must update its own position information according to the formula, put the position information into the matrix *A*_*nm*_ for execution, and calculate the fitness value of the whale at this position according to the fitness function *F*_obj_ to find the optimal position is used as the position of the leader whale for the next iteration. Repeat this process until the iteration ends, and finally find the optimal solution of the entire population in the matrix *A*_*nm*_, that is, the best solution to perform scheduling.

## 6. Multiobjective Task Scheduling Strategy Based on Gaussian Whale-Cloud Optimization in Cloud Computing (GCWOAS2)

Our research found that the whale optimization algorithm randomly generates an initial population at the beginning of the iteration, which cannot guarantee the diversity of the population, and will affect the search efficiency of the algorithm to a certain extent. At the same time, the algorithm does not jump out of the local optimal solution, and it is easy to fall into the local optimal when it converges quickly. Therefore, by making the following improvements to the whale optimization algorithm, we propose a multiobjective task scheduling strategy GCWOAS2 based on Gaussian cloud-whale optimization in cloud computing: (1) generate its reverse population while randomly generating the population to increase the diversity of the population. (2) In the search process, an adaptive movement factor is introduced to expand the search range, so that the algorithm jumps out of the local optimum. (3) The normal cloud model is introduced to make the algorithm enhance the global search and jump out of the local optimum.

### 6.1. Initial Population Algorithm Based on Opposition-Based Learning Mechanism

Since the randomly generated population has not been estimated, it has greater uncertainty, and the convergence rate is unpredictable, so we consider the reverse individual of each individual at the same time, and choose the minimum fitness function value shown in formula (10) as the initial individual, each individual in the population will be closer to the optimal solution, so in the initialization phase, we introduced a reverse learning mechanism to initialize the population.

The pseudocode of the initial population algorithm based on opposition-based learning mechanism is shown in Algorithm 2.

### 6.2. The Formula of Dynamically Adjusting the Moving Step Length Based on the Adaptive Moving Factor

As shown in the above content, formula (13) is mainly used to calculate the encircling step size $\overset{⟶}{A}\times \overset{⟶}{C}$ of the WOA algorithm, and the encircling step size can affect the search range of the algorithm. When placed in the task scheduling of cloud computing, it can intuitively affect the range of tasks to search the suitable VM. It can be seen from formula (14) that the change of $\overset{⟶}{C}$ is directly related to the change of $\overset{⟶}{r_{2}}$, so we propose to improve $\overset{⟶}{r_{2}}$ according to formula (21) as follows:

(24) $$\begin{array}{c} \overset{⟶}{r_{2}}=\tan\left(\frac{\pi }{4}\times \frac{t}{\text{Maxiter}}\right), \end{array}$$

where *t* represents the current iteration number of the population, and Maxiter represents the maximum iteration number of the population. We can see that $\overset{⟶}{r_{2}}$ gradually becomes larger as the number of iterations increases, and $\overset{⟶}{A}$ also gradually becomes larger, and the moving step size increases accordingly. In the initial stage of the search, we can find that the step size is small, which can effectively limit the global search in the population and maintain the diversity of the population; in the later stage of the search, the step size starts to increase gradually, and the global search ability is enhanced, which is convenient for the algorithm to jump out of the local area. So that the global optimal solution can be found.

In addition, we also used the Gaussian cloud model to improve the whale's swimming process to further improve the algorithm's search ability in cloud task scheduling.

### 6.3. Whale Optimization Algorithm Based on Gaussian Cloud Model

The Gaussian cloud model is introduced into the whale optimization algorithm to improve the walking step length of the whale and optimize the random swimming around the prey during the swimming search and the process of driving the prey by the bubble net. Due to the randomness of the Gaussian cloud model, tasks can be matched to more different types of VM in the cloud computing task scheduling process, which expands the search scope, improves the probability of matching to different VMs, and improves the convergence speed. The global optimization capability has also been effectively improved.

#### 6.3.1. Random Walk Process When Surrounding Prey

In order to make the algorithm expand the search range out of the local optimum during the search process, we use the randomness of the Gaussian cloud model to update the position in the random walk process, and the process is as follows:

(25) $$\begin{array}{c} E_{x}=X_{i}, \end{array}$$

(26) $$\begin{array}{c} E_{n}=\frac{\left|X_{b}-X_{w}\right|}{3}, \end{array}$$

(27) $$\begin{array}{c} H_{e}=\frac{E_{n}}{r_{2}}, \end{array}$$

(28) $$\begin{array}{c} E_{n}^{\prime }=N\cdot \left(E_{n},H_{e}\right), \end{array}$$

(29) $$\begin{array}{c} \mu =e^{-\left({\left(X_{i}-E_{x}\right)}^{2}/2{\left(E_{n}^{\prime }\right)}^{2}\right)}, \end{array}$$

where *X*_*i*_ represents the current position, *E*_*x*_ represents the expectation of the Gaussian cloud model, *X*_*b*_ and *X*_*w*_ represent the most and worst positions of the whale, respectively, *E*_*n*_ is the entropy in the Gaussian cloud model, *H*_*e*_ represents the superficial value in the Gaussian cloud model, *E*_*n*_′ represents a random value between entropy and superentropy, and *μ* represents the value of the membership function.

The new position obtained after random walk is shown in the following formula:

(30) $$\begin{array}{c} \overset{⟶}{X}\left(t+1\right)=\mu \times \left(\overset{⟶}{X_{\text{rand}}}\left(t\right)-\overset{⟶}{A}\times \overset{⟶}{D}\right). \end{array}$$

#### 6.3.2. Bubble Net to Drive Away Prey

Here, we propose a method of using the randomness of the Gaussian cloud model to improve the spiral update method of the bubble net in the process of driving prey, increase the choice of the spiral path of the whale in the optimization process, and make the whale have a certain degree in the search process. The randomness increases the search range of the whale group and improves the global search ability of the algorithm:

(31) $$\begin{array}{c} E_{x}=\frac{\left(ub-lb\right)}{20}\times {\left(\frac{1-t}{\text{Iters}}\right)}^{2}, \end{array}$$

(32) $$\begin{array}{c} E_{n}=\text{abs}\frac{\left(X_{b}-X_{w}\right)}{3}, \end{array}$$

(33) $$\begin{array}{c} H_{e}=\frac{E_{n}}{3}, \end{array}$$

(34) $$\begin{array}{c} E_{n}^{\prime }=\text{normrnd}\left(E_{n},H_{e}\right), \end{array}$$

(35) $$\begin{array}{c} w=\text{rand}\left(1\right)\times E_{n}^{\prime }, \end{array}$$

(36) $$\begin{array}{c} S=\exp\frac{\left(-{\left(w-E_{n}\right)}^{2}\right)}{\left(2\times E_{n}^{\prime 2}\right)}. \end{array}$$

In this step, the parameters in the formula have the same meaning as in the previous text, and *S* represents the value of the membership function in the Gaussian cloud model.

Then, the bubble net drives the prey to update the individual position as shown in

(37) $$\begin{array}{c} \overset{⟶}{X}\left(t+1\right)=S\times \left(\overset{⟶}{D^{\prime }}\times e^{Sbl}\times \;\cos\left(2\pi l\right)+S\times \overset{⟶}{X_{b}}\left(t\right)\right). \end{array}$$

According to the basic Gaussian cloud model shown in Algorithm 1, the Gaussian cloud model is introduced into the whale swimming process, and a whale optimization algorithm based on the Gaussian cloud model is proposed, as shown in Algorithm 3.

### 6.4. Multiobjective Task Scheduling Algorithm-GCWOA Algorithm Based on Gaussian Whale-Cloud Optimization in Cloud Computing

**Figure 3:**
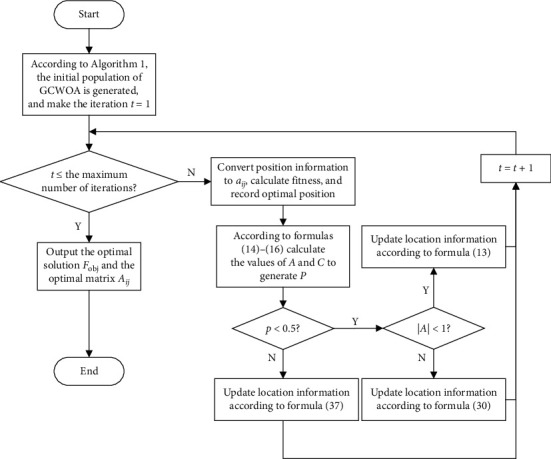
GCWOA algorithm flowchart.

Based on the above design, we propose a multiobjective task scheduling algorithm based on Gaussian whale-cloud optimization (GCWOA) for cloud computing task scheduling, and the flowchart is shown in Figure 3. Its main realization can be divided into the following processes:

- Step 1: this step is mainly used to design the mapping relationship between tasks and whales in cloud computing, and the initial position of each whale. In addition, we also initialize the dimensions of the search space, the upper and lower bounds of the search space, and the maximum number of iterations of the population.
- Step 2: introduce a reverse learning mechanism to generate a suitable scheduling plan in the process of generating the initial population, and expand the search range.
- Step 3: after the initialization is completed, the algorithm begins to iterate. In this step, the optimal position and the worst position of the whale are calculated according to the fitness function we proposed to calculate the time, load, and cost (refer to formula (10)), that is, the best scheduling plan and the worst plan in the scheduling process. At the same time, the coefficient vector is calculated according to the adaptive adjustment factor.
- Step 4: determine the swimming mode of the whale according to the vector $\overset{⟶}{A}$, and guide the whale to randomly swim to find the optimal position according to the process in Algorithm 3, or spiral search to update the position.
- Step 5: when all the whale positions are updated, that is, all scheduling plans are found, the next iteration will be performed. When the maximum number of iterations is reached, the algorithm terminates, otherwise go to Step 2 to continue to update the position of the whale. After the iteration is completed, the optimal position, that is, the most suitable task and virtual machine mapping, will be transferred to matrix A to obtain the best task scheduling plan.

According to the above steps, the complete GCWOA algorithm we get is shown in Algorithm 4, and the algorithm flow is shown in GCWOA.

## 7. Simulation Results and Analysis

In this part, we performed simulation verification and performance evaluation on the GCWOA algorithm proposed in this article and compared it with other existing algorithms. In this experiment, we used CPU (AMD quad-core, 2.4 GHz), memory (4.0 GB), Windows 10 operating system, and MatlabR2016b as the simulation platform and carried out the proposed GCWOA algorithm with common metaheuristic algorithms such as ACO and PSO. For comparison, the algorithm is compared with the original WOA algorithm described in the article. According to our simulation results, we compare and evaluate the performance of these algorithms in cloud computing task scheduling.

### 7.1. Experimental Data Settings and Indicators

The performance evaluation indicators of our algorithm include time, virtual machine load, virtual machine cost, and the total cost of the final model. Our experiment mainly evaluates the performance of each algorithm in a small number of tasks. The main parameters used by each algorithm in the experiment are shown in Table 1, and the VM and task parameters are shown in Table 2. In addition, we set the number of VMs to 40, and the number of search agents for all algorithms is set to 50 (that is, there are 50 sets of solutions). As the number of iterations increases, the algorithm will get closer and closer to the optimal solution, which is of great significance to cloud computing task scheduling [48], so we set the number of iterations to 100.

### 7.2. Experimental Results and Analysis

We implement and evaluate the performance of each algorithm by increasing the number of iterations and changing the number of tasks. The experimental result table is shown in Table 3. From Table 3, we can see the value of the fitness function of each algorithm when iterating 100 times and the number of tasks is 100 and 1000.

**Figure 4:**
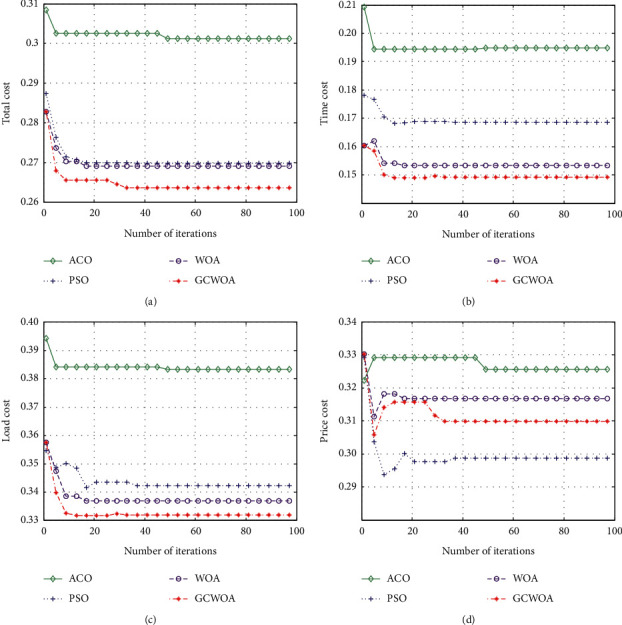
The best performance of 100 tasks with the number of iterations. (a) The value of total cost. (b) The value of time cost. (c) The value of load cost. (d) The value of price cost.

In Figure 4, we can see that as the number of iterations increases, the GCWOA algorithm is better than other algorithms in terms of total cost, which effectively proves the role of our algorithm in the task scheduling process. Since the opposition-based learning mechanism is introduced when the GCWOA algorithm initializes the population, the GCWOA algorithm is closer to the optimal value than other algorithms at the beginning of the iteration. The existence of the adaptive adjustment factor makes the algorithm search a larger range in the search process, so as to find more different solutions than other algorithms. The introduction of the Gaussian cloud model enables the GCWOA algorithm to jump out of the local optimal and find the global optimal solution. In addition, we can see that the optimization effect of the GCWOA algorithm we proposed is the best. Compared with the three algorithms of WOA, PSO, and ACO, the total cost of the GCWOA algorithm is reduced. At the same time, we can see that the GCWOA algorithm converges rapidly at the beginning of the iteration, and has stabilized at the 16th iteration, indicating that the GCWOA algorithm can converge to the optimal solution relatively quickly.

The time cost and virtual machine load are shown in Figures 4(b) and 4(c). In these two figures, the values of the four algorithms are decreasing with the increase of the number of iterations, indicating that the four algorithms are searching in the process, and costs can be effectively reduced. It is worth mentioning that the GCWOA algorithm has much better performance than the other three algorithms in terms of convergence accuracy.

However, compared with Figures 4(a)–4(c) in Figure 4, the virtual machine cost shown in Figure 4(d) is different. Although the value of the four algorithms in processing virtual machine costs fluctuates with the increase in the number of iterations, from a global perspective, the PSO, WOA, and GCWOA algorithms all decrease significantly and then increase significantly until they reach a stable state.

This shows that these three algorithms consume a large amount of virtual machine resources at a certain stage. In general, the GCWOA algorithm does not optimize this cost well. The reason for this problem may be due to multiobjective scheduling. In order to achieve the global optimal solution, the GCWOA algorithm sacrifices a certain local optimal value (here, the virtual machine cost) in the search process to balance the two. At the same time, it also proves that the GCWOA algorithm can be used to solve complex scheduling problems.

**Figure 5:**
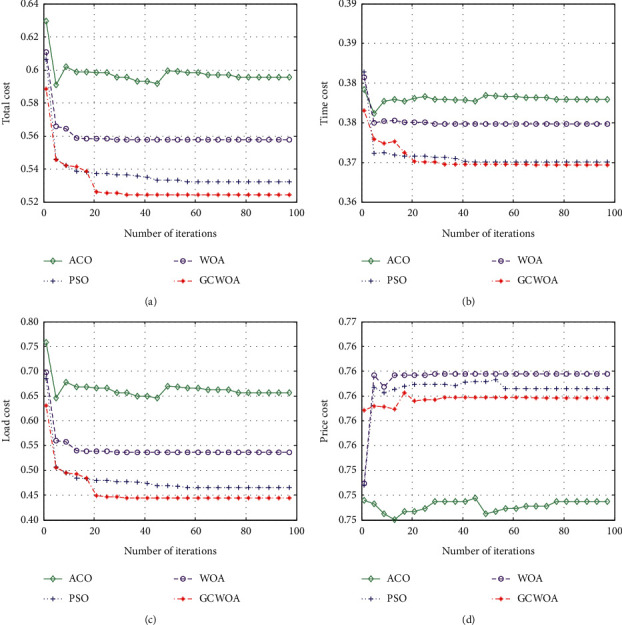
The performance of 1000 tasks with the number of iterations. (a) The value of total cost. (b) The value of time cost. (c) The value of load cost. (d) The value of price cost.

We also evaluated the performance of the four algorithms with the number of iterations under large-scale tasks. As shown in Figure 5(a), although the total cost of the four algorithms has increased compared with the amount of 100 tasks, the total cost of each algorithm decreases as the number of iterations increases, which shows the effectiveness of the algorithm in cloud computing task scheduling. In addition, the algorithms converge quickly at the beginning of the iteration. The GCWOA algorithm continues to converge until it stabilizes at the 20th iteration, indicating that the algorithm has jumped out of the local optimum and found the most suitable scheduling scheme. It also proves that the strategy we proposed is effective.

Compared with the other three algorithms, the total cost of our proposed GCWOA algorithm is significantly lower than other algorithms, which proves that the GCWOA algorithm can also optimize large-scale task scheduling. The time cost and load cost of the four algorithms are shown in Figures 5(b) and 5(c). We can see that the GCWOA algorithm can more effectively use resources when processing large-scale tasks and reduce task execution time. At the same time, it reduces resource consumption, saves memory, and reduces the underlying load cost. In the iterative process, the algorithm converges rapidly. After reaching the local optimum, the curve continues to decrease until it is stable, which reflects the advantages of the GCWOA algorithm in the scheduling of fast convergence and easy to jump out of the local optimum.

As shown in Figure 5(d), the virtual machine operating cost is different when processing large-scale tasks and small-scale tasks. Although the cost of GCWOA is still higher than other algorithms, when we consider the total cost, the GCWOA algorithm is still superior to other algorithms and has a stronger global search capability than other algorithms.

## 8. Conclusion

The rapid increase in the number of user tasks has led to data flooding, low scheduling efficiency, and unbalanced resource allocation, making task scheduling in cloud computing become an agenda. This paper proposes a “whale-Gaussian cloud” model, which combines heuristic algorithms and Gaussian cloud to achieve better scheduling. On the basis of this model, we propose a scheduling algorithm based on GCWOA. In addition to combining the whale optimization algorithm with the Gaussian cloud model, we also introduce an opposite learning mechanism and an adaptive adjustment factor into the algorithm. The opposite learning mechanism is used to generate more suitable task-VM mapping. Both the adaptive adjustment factor and the Gaussian cloud model can guide the algorithm in the whale optimization algorithm to select the optimal scheduling scheme in cloud computing. We have conducted a large number of experiments to verify the performance of our proposed scheduling strategy. The experimental results show that the algorithm proposed in this paper has ideal effects in processing small tasks and large tasks and can reduce task completion time and balance load of virtual machines.

However, no matter how large the task is, the algorithm in this paper does not perform well in terms of operating costs. How to make the GCWOAS2 strategy further reduce operating costs will be the focus of our future research. In the future, we will continue to study the application of heuristic algorithms in multiobjective scheduling to enable more effective and reasonable scheduling of resources in cloud computing nodes.

## Figures and Tables

**Algorithm 1:**
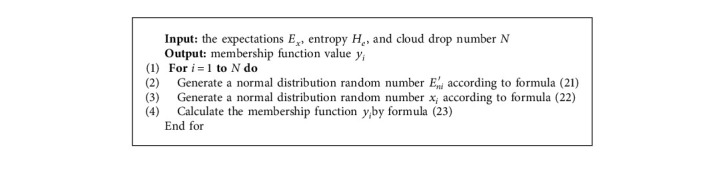
Gaussian cloud model generation.

**Algorithm 2:**
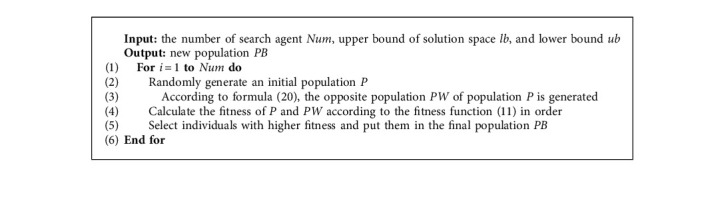
Population initialization based on OBL.

**Algorithm 3:**
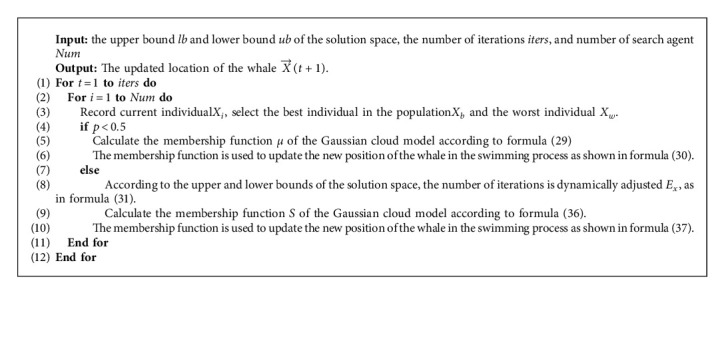
Whale optimization algorithm based on the Gaussian cloud model.

**Algorithm 4:**
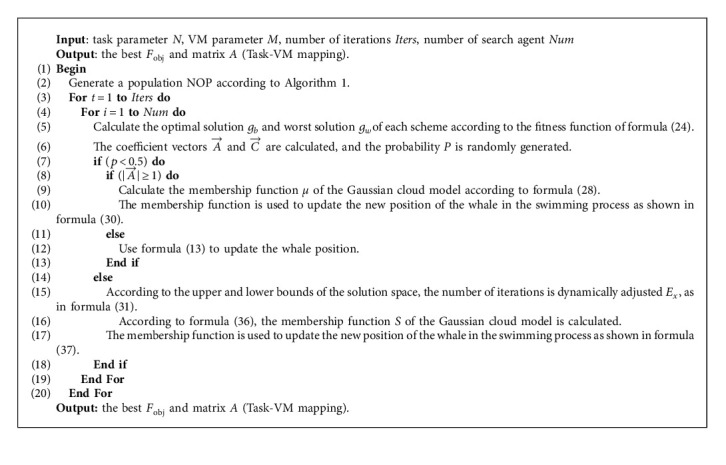
Multiobjective task scheduling algorithm based on the Gaussian whale-cloud optimization-GCWOA algorithm.

**Table 1:** The main parameters of the algorithm.

Algorithm	Parameter	Value	Description
ACO	*ρ*	0.7	Pheromones
	*p*	0.3	Probability of path selection

PSO	*ω*	0.9	Inertia weight
	*c*_1_	1.8	Acceleration constant
	*c*_2_	1.8	Acceleration constant

GCWOA(WOA)	*b*	1	Spiral search path parameters
	*lb*	−5	Upper bound of solution space
	*ub*	10	Lower bound of solution space

**Table 2:** VM and task parameters.

Parameter	Value range
CPU (VM)	[200, 500]
Memory (VM)	[100, 500]
Bandwidth (VM)	[100, 250]
Length (Task)	[50, 100]

**Table 3:** Experimental results after 100 iterations.

Number of tasks	Target value	Algorithm			
		ACO	PSO	WOA	GCWOA
Task100	F1	0.3012	0.2698	0.2690	0.2623
	F2	0.1948	0.1685	0.1533	0.1489
	F3	0.3833	0.3424	0.3370	0.3302
	F4	0.3255	0.2986	0.3168	0.3098

Task1000	F1	0.5954	0.5322	0.5576	0.5245
	F2	0.3779	0.3701	0.3749	0.3684
	F3	0.6569	0.4659	0.5362	0.4440
	F4	0.7515	0.7606	0.7618	0.7599

## Data Availability

The data used to support the findings of this study are available from the corresponding author upon request.
